# A perspective on therapies for amyotrophic lateral sclerosis: can disease progression be curbed?

**DOI:** 10.1186/s40035-021-00250-5

**Published:** 2021-08-10

**Authors:** Xiaojiao Xu, Dingding Shen, Yining Gao, Qinming Zhou, You Ni, Huanyu Meng, Hongqin Shi, Weidong Le, Shengdi Chen, Sheng Chen

**Affiliations:** 1 School of Medicine, University of Electronic Science and Technology of China, Chengdu, 610054 China; 2 Department of Neurology, Ruijin Hospital, Shanghai Jiaotong University School of Medicine, Shanghai, 200020 China; 3 Department of Neurology, Xinrui Hospital, Wuxi, 214028 China; 4 Institute of Neurology, Sichuan Academy of Medical Sciences-Sichuan Provincial Hospital, Chengdu, 610031 China; 5 Center for Clinical Research on Neurological Diseases, the First Affiliated Hospital, Dalian Medical University, Dalian, 116021 China

**Keywords:** Amyotrophic lateral sclerosis, Motor neurons, Autophagy, Stem cells, Gene editing

## Abstract

Amyotrophic lateral sclerosis (ALS) is a progressive neurodegenerative disease involving both upper and lower motor neurons, leading to paralysis and eventually death. Symptomatic treatments such as inhibition of salivation, alleviation of muscle cramps, and relief of spasticity and pain still play an important role in enhancing the quality of life. To date, riluzole and edaravone are the only two drugs approved by the Food and Drug Administration for the treatment of ALS in a few countries. While there is adequate consensus on the modest efficacy of riluzole, there are still open questions concerning the efficacy of edaravone in slowing the disease progression. Therefore, identification of novel therapeutic strategies is urgently needed. Impaired autophagic process plays a critical role in ALS pathogenesis. In this review, we focus on therapies modulating autophagy in the context of ALS. Furthermore, stem cell therapies, gene therapies, and newly-developed biomaterials have great potentials in alleviating neurodegeneration, which might halt the disease progression. In this review, we will summarize the current and prospective therapies for ALS.

## Introduction

Amyotrophic lateral sclerosis (ALS), also known as “Lou Gehrig’s disease”, is a progressive neurodegenerative disease that affects both upper and lower motor neurons (MNs), resulting in paralysis and eventually death generally due to respiratory failure [1–3]. ALS is a rare disease with a reported incidence between 0.8–3.6 per 100,000 people year, and significant geographical heterogeneity exists [4, 5]. The median survival time of ALS patients is 3–5 years since disease onset [3, 6]. Increasing age and male gender are two major risk factors for ALS, thus people in the late 50s and men are more susceptible, with a male-to-female ratio of 1.5:1 for ALS incidence [7, 8]. Apart from age and gender, trauma, cigarette smoking, alcohol consumption, a high fat intake, high levels of premorbid fitness, environmental exposure to heavy metals, pesticides and chemicals, and electric shocks have been reported to be associated with an increased risk of ALS [3, 9–11]. Occupation and education are other two frequently studied factors in ALS. It has been suggested that a low level of education and occupations including veterinarian, athlete, hairdresser, craft and related trades worker, and armed forces personnel might increase the ALS risk [9, 12].

Although the clinical hallmark of ALS is progressive motor deficit, the initial symptoms depending on the degree of involvement of upper and lower MNs vary among patients [1]. Approximately 60% of cases are limb-onset, and symptoms are usually asymmetrical in presentation and can first develop in the upper or lower limbs, with muscle weakness and atrophy. Bulbar-onset disease, which is featured by dysarthria and dysphagia, accounts for one-third of ALS patients [13, 14]. The clinical heterogeneity of ALS and the lack of biological diagnostic markers have hindered or delayed appropriate diagnosis. The El Escorial criteria, the consensus guidelines for the diagnosis of ALS which were first established in 1994 and revised in 2000 by Brooks, have been widely accepted [15, 16]. In this criteria, clinical examination, nerve conduction, electromyography, and laboratory test data are combined to exclude other possible diagnoses. Additionally, the Revised Amyotrophic Lateral Sclerosis Functional Rating Scale (ALSFRS-R), a validated clinical questionnaire-based scale, has been formulated to measure ALS patients’ physical function in carrying out activities of daily living, with a lower score usually predicting a poorer prognosis. The ALSFRS-R has been widely used in clinical trials to track progression of the disease and assess the efficacy of drugs [17].

To date, disease-modifying therapies for ALS remain restricted to two drugs, riluzole and edaravone, and they provide only modest clinical benefits [1]. Apart from them, multiple therapies targeting possible disease mechanisms or directly targeting disease-causing genes have been tested in ALS patients or mouse models, showing great potential for future clinical use. The clinical trials of these therapies in ALS are summarized in Table 1. In this article, we will review the recent advances in potential therapeutic strategies for ALS, with a sense of optimism that significant survival improvement could be expected in the future.

**Table 1:** Clinical trials in recent 10 years

Clinical trial identifier	Phase /subjects	Drug	Treatment	Duration	Primary outcome measure	Main findings
***Oxidative stress***						
NCT00330681/ [18]	Phase III 206 participants	Edaravone (MCI-186)	Placebo (*n* = 104) or edaravone (*n* = 102) i.v. infusion over 60 min for the first 14 days in cycle 1, and for 10 of the first 14 days during cycles 2 to 6.	2006.05-2008.09	ALSFRS-R	Small reduction of ALSFRS-R scores was observed in the edaravone group.
NCT01492686/ [19]	Phase III 137 participants	Edaravone (MCI-186)	Placebo (*n* = 69) or edaravone (*n* = 68) i.v. infusion over 60 min for the first 14 days in cycle 1, and for 10 of the first 14 days during cycles 2 to 6.	2011.12–2014.9	ALSFRS-R	Edaravone improved ALSFRS-R scores in a small subset of people.
***Autophagy***						
UMIN000036295/ [20]	Phase I	Bosutinib	Three to six patients with ALS were enrolled at each of the four bosutinib dose levels (100, 200, 300 or 400 mg/day).	2019.03–2021.03	DLT	Undergoing.
NCT02166944/ [21]	Phase I/II 20 participants	Tamoxifen	Tamoxifen 40 mg (*n* = 10) or riluzole (*n* = 8) daily for 1 year.	2014.04–2019.09	ALSFRS-R	Tamoxifen exerted only a modest effect in attenuating progression for 6 months.
***Cell therapy***						
NCT01640067/ [22]	Phase I 18 participants	HSSCs	Three received 3 unilateral injections of hNSCs into the lumbar cord tract, while the others received bilateral injections. A total of 750,000 cells per injection site (15 μ).	2011.12–2015.12	Treatment-related mortality, AEs, neuroradiological and neurophysiological variables	Transplantation of hNSCs was confirmed to be a safe cell therapy approach with good reproducibility. Transient improvement in ALSFRS-R and MRC was observed in some patients.
NCT01348451/ [23]	Phase I 18 participants	HSSCs	Ten microliters were delivered at a rate of 5 μl/min over 2 min by unilateral cervical injections, for a total of 500,000 cells (NSI-566RSC HSSC line) in the 5 injections.	2009.01–2016.12	AEs	Safety and feasibility of cervical and dual-targeting approaches (both lumbar and cervical injection) was demonstrated.
NCT01730716/ [24]	Phase II 18 participants	HSSCs	Three participants in each group. The numbers of injection (site: C3-C5 or L2-L4 bilateral injections) ranged from 10 to 40, and the numbers of cells (HSSCs) injected ranged from 2 million to 16 million.	2013.05–2016.11	AEs	Intraspinal transplantation of HSSCs was safe at high doses (20 injections, 400,000 cells/injection), including successive lumbar and cervical injections.
NCT01640067/ [25]	Phase I 18 participants	hNSCs	Participants were divided into 3 groups with monolateral or bilateral injections (C3-C5 or T8-T11) of a total of 750,000 cells (15 μ hNSCs) per injection.	2011.12–2015.12	Treatment-related mortality, AEs, neuroradiological and neurophysiological variables	Safety of hNSC transplantation was confirmed. A transitory decrease in progression of ALSFRS-R was observed, starting within the first month after surgery and up to 4 months after transplantation.
NCT01363401/ [26, 27]	Phase I/II 72 participants	BM- MSCs	Each participant received 2 intrathecal injections of autologous BM-MSCs (1 × 10^6^ cells/kg) 26 days apart. Control group (*n* = 31, riluzole 100 mg alone).	2011.02–2013.08	ALSFRS-R	Two repeated intrathecal injections were safe and feasible throughout the 12-month duration.
NCT01051882/ [28]	Phase I/II 12 participants	NurOwn®	Six patients in each group received i.m. or i.t. injection of NurOwn®.	2011.06–2013.03	Safety evaluation and tolerability.	Safe and well-tolerated.
NCT01777646/ [28]	Phase IIa 14 participants	NurOwn®	Fourteen patients received combined i.t. and i.m. delivery. (IM at 24 sites to the biceps and triceps (1 × 10^6^ cells/site); i.t. of 1 × 10^6^ cells/kg)	2012.12–2015.09	Safety evaluation and tolerability	Improvement in the decrease rate of progression of the FVC and ALSFRS-R was demonstrated in the i.t. (or i.t. + i.m.)–treated groups.
NCT02017912/ [29]	Phase II 48 participants	MSC-NTF cells	MSC group (*n* = 36): MSC-NTF cells. Placebo (*n* = 12): Dulbecco Modified Eagle Medium. Combination of i.t. (125 × 10^6^ cells) and 24 i.m. (48 × 10^6^ cells) injections of NurOwn® at 24 sites to the biceps and triceps	2014.05–2016.07	AEs	In a prespecified rapid progressor subgroup (*n* = 21), the rate of disease progression was improved at early time points.
NCT02286011/ [30]	Phase I 20 participants	MNC of BM	Experimental group: an intramuscular infusion of autologous MNC of bone BM in TA muscle of one of the lower limbs (100–1200 million) diluted in 2 ml saline.	2014.11–2017.12	AEs	The intramuscular injection of BMMCs was safe and had an effect on the D50 index.
NCT03241784/ [31]	Phase I 4 participants	Autologous T-regulatory lymphocytes	A total of 8 infusions of autologous Tregs (1 × 10^6^ cells/kg) with concomitant subcutaneous IL-2 injections (3 times /week, 2 × 10^5^ IU/m^2^/injection).	2016.05–2018.02	AEs	The numbers of Tregs and suppressive function increased after infusion and the increased suppressive function of Tregs correlated with slowing of progression rate.
***Gene modification***						
NCT01041222/ [32]	Phase I 33 participants	ISIS 333611	Four cohorts of eight patients received intrathecal infusion of ISIS 333611 at dose of 0.15 mg, 0.5 mg, 1.5 mg, 3 mg, respectively (randomized 6 drugs: 2 placebo/cohort).	2010.01–2012.01	Safety, pharmacokinetics tolerability	No dose-limiting toxicity was found at doses up to 3.0 mg. Dose-dependent CSF and plasma concentrations were observed.
NCT02623699/ [33]	Phase I/II 84 participants	Tofersen (BIIB067)	In each dose cohort (20, 40, 60, or 100 mg), participants were randomly assigned in a 3:1 ratio to receive five doses of tofersen or placebo, administered intrathecally for 12 weeks.	2016.01–2019.01	AEs	CSF SOD1 concentrations decreased at the highest concentration of tofersen administered intrathecally over a period of 12 weeks.
***Excitotoxicity***						
NCT00444613/ [34]	Phase II/III 373 participants	Mecobalamin (E0302)	Placebo (*n* = 124), 25 mg (*n* = 124) or 50 mg methylcobalamin (*n* = 125) administered intra-muscularly twice a week for 182 weeks.	2007.04–2014.03	Survival rate, ALSFRS-R	No significant efficacy was seen in the whole cohort. The treatment may prolong survival and retard symptomatic progression if started early (≤12 months’ duration).
***Mitochondrial defects and apoptosis***						
NCT01786603/ [35]	Phase II 80 participants	Rasagiline	Rasagiline group (*n* = 60): rasagiline 2 mg P.O. once a day for 12 months. Placebo group (*n* = 20): placebo 2 mg once a day for 12 months.	2013.11–2016.07	ALSFRS-R	Rasagiline was well tolerated with no serious adverse events. No improvement in the ALSFRS-R slope was observed in the rasagiline group.
NCT01879241/ [36]	Phase II 252 participants	Rasagiline	Rasagiline group (*n* = 127): 100 mg riluzole plus 1 mg rasagiline P.O.Placebo group (*n* = 125): 100 mg riluzole plus placebo P.O. per day for 18 months.	2013.06–2016.08	Survival	Disease progression might be modified by rasagiline in patients with normal to fast progression rate, despite no efficacy in survival.
***Immunomodulatory***						
NCT02588677/ [37]	Phase II/III 394 participants	Masitinib	394 patients were randomly assigned (1:1:1) to receive riluzole (100 mg/d) plus placebo or masitinib at 4.5 or 3.0 mg/kg per day.	2013.04–2018.03	ALSFRS-R	Masitinib showed significant benefits over placebo with a between-group difference in △ALSFRS-R, corresponding to 27% slowing in the rate of functional decline.

*AEs* adverse events, *ALSFRS-R* revised ALS functional rating scale, *DLT* dose-limiting toxicity, *i.v.* intravenous, *HSSC* human spinal stem cells, *hNSCs* fetal human neural stem cells from natural in utero death, *BM-derived MSCs* bone marrow-derived mesenchymal stem cells; *i.m.* intramuscular, *i.t.* intrathecal, *MSC-NTF* mesenchymal stem cells-neurotrophic factors, *VC* vital capacity, *CSF* cerebrospinal fluid, *ISIS 333611* an antisense oligonucleotide designed to inhibit SOD1 expression, *MNC* mononuclear cells, *BM* bone marrow

## Genes and pathogenic mechanisms of ALS

About 60% of the risk of ALS can be attributed to the genetics and ALS can be categorized into familial (fALS) and sporadic ALS (sALS) [9, 38], which account for 10% and 90% of ALS cases, respectively [1]. More than 30 genes have been identified in fALS [8, 39], of which mutations in genes encoding superoxide dismutase 1 (*SOD1*), chromosome 9 open reading frame 72 *(C9orf72)*, TAR DNA binding proteins (*TARDBP*/*TDP43*) and fused in sarcoma (*FUS*) account for at least 50% of all fALS cases [14, 40]. Mutations in *SOD1*, the first identified gene in fALS, occur in up to 20% of fALS cases and 1%–4% of sALS cases. SOD1^G93A^ mice are the most commonly used model in ALS studies investigating molecular mechanisms and evaluating drug efficacy [41].

Although the precise mechanisms underlying ALS remain unclear, many pathologic processes have been implicated, including glutamate excitotoxicity, protein misfolding and aggregation, impaired protein degradation (involving impairment of autophagy and proteasome), oxidative stress, axonal transport abnormalities, inflammation, aberrant RNA metabolism, mitochondrial dysfunction, and endoplasmic reticulum (ER) stress [1, 13, 14, 42].

Genetic polymorphism can potentially modify drug response, an effect named pharmacogenomics. In an analysis of genetic interaction between three common ALS-related genes and creatine monohydrate and valproic acid treatment in two clinical trials, a dose-response pharmacogenetic interaction between creatine and the A allele of the MOBP genotype (rs616147) was identified, highlighting the importance of incorporating genetic information in ALS clinical trials [43].

## Current clinical treatments

Over the past two decades, more than 50 drugs have shown efficacy in extending life expectancy in preclinical animal models of ALS [14]. Yet, there is still no cure for ALS that could reverse the progression of this disorder from a clinical perspective. Riluzole and edaravone are the only two disease-modifying drugs for the treatment of ALS [1, 13, 44, 45].

### Riluzole

Riluzole is a glutamate release inhibitor that blocks voltage-gated sodium channels, and is the first drug approved by FDA for ALS treatment in 1995 [1]. So far, there has been adequate consensus on the modest efficacy of riluzole. Recently, reanalysis of case records of all 959 participants from a previous dose-ranging trial showed that higher-dose riluzole (100 mg/day) prolonged stage 4 in patients with ALS instead of slowing the entire disease course or prolonging stage 2 or 3 [46]. Results from other trials have suggested greater benefits from riluzole treatment at early stage before the occurrence of significant degeneration of MNs [47, 48]. Fan et al. have investigated the effect of daily and cumulative riluzole in a long-term follow-up of an ALS cohort in China [49] and reported a better prognosis in patients receiving the cumulative defined daily dose of riluzole higher than 16,800 mg than other groups, highlighting the importance of long-term riluzole use.

### Edaravone

Edaravone is a free radical scavenger that has been approved for the treatment of ALS in a few countries (approved in Japan in 2015, South Korea in 2015, the United States in 2017, Canada in 2018, Switzerland in 2019, China in 2019, and Indonesia in 2020) [44, 50]. Edaravone has demonstrated effects in reducing oxidative stress and delaying functional motor deterioration in a previous clinical study [51]. However, a phase III confirmatory trial (MCI 186–16) has failed to demonstrate the efficacy of edaravone in the prolongation of survival and respiratory function improvement [18]. Subsequent post-hoc analysis of the MCI 186–16 trial revealed a positive effect of edaravone in a restricted subgroup with milder symptoms and a disease duration within 2 years, and the effect could last for over a period of 24 weeks [52]. Notably, in a second phase III trial (MCI 186–19, NCT01492686) in which patients were well defined by strict inclusion criteria, the edaravone group showed a significant improvement in the ALSFRS-R score when compared with the placebo group, suggesting a beneficial effect of edaravone for over 24 weeks [19]. However, a latest study in Italian ALS patients has demonstrated no significant difference between Edaravone-treated and control groups in either disease progression or respiratory function [53]. Considering that edaravone has just been approved in certain countries for a few years, and few clinical trials carried out in recent years have demonstrated the beneficial effect of edaravone, the efficacy of edaravone in ALS treatment remains controversial. Hence, further clinical studies with stringent inclusion criteria, long duration, and reliable pathobiological markers are urgently required to validate the efficacy of edaravone.

### Other symptomatic treatments

ALS patients suffer from a wide range of debilitating symptoms, including fatigue, cramps, spasticity, dysphagia, respiratory insufficiency, sleep disorders, pain, psychosocial morbidity, etc. Supportive care plays an important role in providing symptom management and improving quality of life. The symptoms of ALS could be relieved by pharmacological and non-pharmacological interventions. Thus, multidisciplinary ALS care alleviating the symptoms can enhance the quality of life and prolong the survival of patients [14]. Expert consensus guidelines for supportive and symptomatic management of ALS are available [2]. However, high-quality evidence for the effectiveness of symptomatic therapies is very limited. Double-blind, placebo-controlled studies are available for only a few symptomatic treatments, such as botulinum toxin for sialorrhea [54] and dextromethorphan plus quinidine for pseudobulbar affect [55].

Spasticity is a common symptom present in most patients with ALS. Muscle relaxants such as baclofen and tizanidine should only be used for disabling spasticity because of the side effects of aggravation of muscle weakness and the sedating effect [2, 56]. Baclofen could be administered through an intrathecal pump if oral administration is ineffective [57]. Additionally, cannabinoids may have potential value in the treatment of spasticity in ALS patients, as they can control spasticity with safety and efficacy in multiple sclerosis [58, 59]. Most recently, a placebo-controlled phase II trial has demonstrated that nabiximols, a combination of tetrahydrocannabinol and cannabidiol, have a positive antispastic and pain-relieving effect in patients with motor neuron disease [60]. Furthermore, a retrospective mono-centric cohort study has revealed high treatment satisfaction with nabiximols, suggesting that nabiximols are an addition to ALS symptomatic therapy [61]. Preclinical studies have also shown neuroprotective effects of cannabinoids, such as reducing excitotoxicity and oxidative damage, and suppressing neuroinflammation and microglial activation by activating CB1 and particularly CB2 receptors. Besides, application of cannabinoids can delay motor impairment and prolong survival in murine models [62–64]. Taken together, these data demonstrate the great potential of cannabinoids in ALS as a supplement therapy.

Respiratory failure is the leading cause of death in ALS. Non-invasive ventilation has shown benefits of prolonging survival by 7 months and improving the quality of life in an earlier small randomized controlled trial (RCT) including 41 ALS patients [65], and the FDA approved diaphragmatic pacing as a weakened diaphragm stimulator for ALS treatment in 2011 [2]. However, a more recent multicenter, open-label RCT (ISRCTN 53817913) demonstrated that addition of diaphragm pacing to standard care with non-invasive ventilation was associated with decreased survival and should not be used as a treatment for ALS patients [66]. Similar results were reported in another multicenter trial (NCT01583088) [67]. Recently, mexiletine has been tested for its efficacy in ALS due to its effect in reducing muscle cramps in Machado-Joseph disease. Although results showed no effect on the functional disability, impairment and survival in ALS, mexiletine induced a significant improvement in muscle cramp severity and frequency [68, 69].

Weight loss and malnutrition are common features of ALS. Reduced survival time and poor quality of life correlate with the nutritional status of ALS patients [70]. The European Federation of Neurological Societies guidelines recommend gastrostomy for ALS patients with severe dysphagia when weight loss reaches at least 10% from premorbid weight. A recent large, longitudinal, prospective cohort study enrolling 345 ALS patients has shown no difference in the safety regarding survival and procedural complications among three methods of gastrostomy, and that the median survival (12 months) after gastrostomy for patients with weight loss of 10% or less from that at diagnosis was increased by about 4 months when compared with those who had lost more than 10% of their weight from diagnosis (7.7 months) [71]. This finding indicates that less than 10% of weight loss might be an optimum timing for gastrostomy insertion to achieve clinical benefits. Yu et al. enrolled 272 Korean sporadic ALS patients to investigate the relationship between dietary fiber intake and the prognosis of ALS. Kaplan-Meier analysis showed a significant distinction in the mean survival time according to vegetable fiber intake, with patients in the highest tertile of vegetable fiber intake showing a longer survival and a lower rate of disease progression. Moreover, the vegetable fiber intake is negatively correlated with the level of pro-inflammatory cytokines (interleukin [IL]-1β, IL-6 and monocyte chemoattractant protein-1) in the cerebrospinal fluid (CSF) [72]. Therefore, dietary fiber supplementation intervention could be included into ALS clinical trials to further validate its efficacy in improving prognosis of ALS.

## Prospective disease-modifying therapies

### Autophagy-targeting therapies

**Fig. 1:**
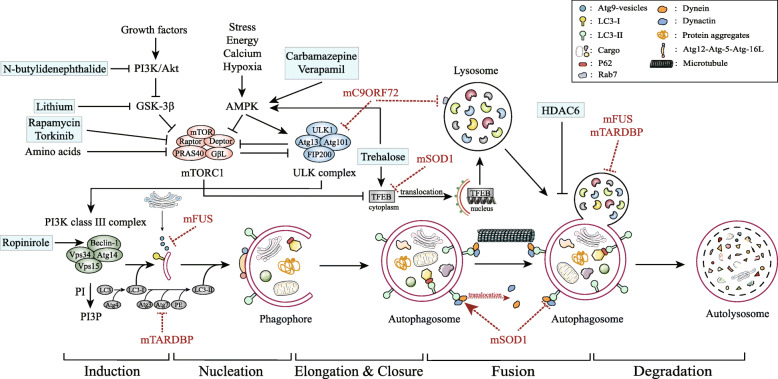
General autophagy process and targets for potential drugs inducing autophagy. Autophagy can be induced by stress, energy deficiency, increased intracellular Ca^2+^, etc., through inhibition of the mTOR complex and subsequent activation of the ULK complex. The class III PI3K complex can be phosphorylated by ULK, subsequently catalyzing PI into PI3P and initiating autophagy. Atg9 vesicles are released from the Golgi complex and recruit the PI3K complex to downstream autophagy-related proteins. The Atg12-Atg5-Atg16L complex and LC3 are ubiquitin ligases that are indispensable for membrane elongation and closure. LC3 can be cleaved by Atg4, and the generated LC3-I binds with PE, which is mediated by the Atg12-Atg5-Atg16L complex, localized on the membranes of autophagosomes. The dynein-dynactin complex mediates the transportation of organelles along the microtubule. Mature vesicles labeled by LC3 are distributed along microtubules and LC3 colocalizes with dynein-dynactin complex. mSOD1 alters the cellular localization of dynein and inhibits the dynein-dynactin complex, impeding the transportation of autophagosomes. TFEB is regulated by mTORC1 to mediate the expression of autophagy and lysosome-related protein (atg9B and LAMP1), which in turn affects the formation of autolysosome. mSOD1 also interferes with the expression of TFEB. Rab7 regulates the formation and maturation of autolysosome, and interacts with *C9ORF72*. Lithium and n-butylidenephthalide enhance autophagy by inhibiting PI3K and GSK-3β, Rapamycin and Torkinib induce autophagy by inhibiting mTORC1, while carbamazepine, verapamil and trehalose initiate autophagy by activating AMPK. Also, trehalose regulates the phosphorylation and translocation of TFEB. It has been reported that ropinirole induces autophagy through a Beclin-1-dependent pathway. HDAC6 can control the fusion of autophagosomes and lysosomes. mTORC1: mechanistic target of rapamycin complex 1, ULK1: unc-51-like kinase 1, AMPK: AMP-activated protein kinase, PI3K: phosphoinositide 3-kinase, GSK-3β: glycogen synthase kinase-3β, PI: phosphatidylinositol, PI3P: phosphatidylinositol-3-phosphate, Atg: autophagy-related protein, LC3: microtubule-associated protein 1A/1B-light chain 3, PE: phosphatidyl ethanolamine, mSOD1: mutant SOD1, TFEB: transcription factor EB, LAMP1: lysosomal-associated membrane protein 1, Rab7: Ras-related protein 7

Autophagy, a catabolic and recycling process that eliminates dysfunctional organelles and abnormal protein aggregates in the cell, is essential to neuronal homeostasis. Impaired autophagy represents a critical pathomechanism in ALS, and autophagic regulation is therefore emerging as a potential therapeutic strategy for ALS (Fig. 1) [73].

#### Rapamycin

The mammalian target of rapamycin (mTOR) pathway is the best-characterized regulator of autophagy initiation. Rapamycin, a widely used autophagy enhancer by inhibiting the mTOR pathway, has presented controversial effects in different genetic animal models of ALS, which limits its practical use in ALS treatment [74–76].

#### Trehalose

Trehalose is a natural disaccharide that induces mTOR-independent autophagy. Administration of trehalose has been shown to prolong the lifespan and delay the disease onset in SOD1^G86R^ mice, accompanied by reduced SOD1 accumulation and enhanced MN survival in the spinal cord [77]. Similar therapeutic effects have been demonstrated in a SOD1^G93A^ mouse model of ALS [78], but the efficacy diminishes during later stages of the disease [79].

#### Lithium

Although effective in delaying the disease onset and extending the lifespan in different ALS mouse models by regulating the mTOR-independent pathway [80–82], lithium failed to show therapeutic benefits in ALS patients in previous clinical trials [83–87]. However, a recent meta-analysis on the different responses between genotypes has found that the treatment effect of lithium is not homogenous across patients. Patients carrying *UNC13A* (protein unc-13 homolog A) mutation could benefit more from lithium treatment than *C9orf72* mutation carriers, with 12-month survival probability improving from 40.1% to 69.7%. This result provides a new insight for future clinical trials and suggests that we should start focusing on genotype-targeted therapies and standardize genotyping due to the heterogeneity of ALS [88].

#### N-butylidenephthalide

N-butylidenephthalide is the main component of a traditional Chinese medicine Danggui. It can regulate autophagy by mediating ER stress [89]. Oral administration of 250 mg/kg (bid) n-butylidenephthalide before the onset of the disease has shown a better effect on survival than riluzole in an ALS mouse model [90]. Furthermore, administration of n-butylidenephthalide could decrease MN loss and restore the gastrocnemius function of SOD1^G93A^ mice. These neuroprotective effects may be associated with the inhibition of autophagy through the Akt/mTOR signaling pathway, and anti-apoptosis, anti-inflammation and antioxidative effects [91].

#### Carbamazepine

Carbamazepine is a well-known anti-epileptic drug that has been reported to stimulate autophagy by decreasing the intracellular level of inositol [92]. Carbamazepine could activate autophagy *via* the mTOR-independent pathway and rescue the motor dysfunction of frontotemporal lobar dementia (FTLD-U) mice with TDP-43 proteinopathies [75]. In SOD1^G93A^ mice, oral administration of carbamazepine (200 mg/kg per day) could delay the disease onset and significantly extend survival. In addition, carbamazepine increases the clearance of mutant SOD1 aggregates *via* the AMPK-ULK1 pathway, which plays a protective role in the preservation of MNs [93].

#### *W. somnifera*

*W. somnifera* is a perennial herb containing the component Withaferin A which has been reported to have a beneficial effect in both TDP-43 and SOD1^G93A^ mouse models [94, 95] and increase autophagosomes [96]. Recent studies have proven that *W. somnifera* has the same effect as Withaferin A and can serve as an autophagy-inducer [97, 98].

#### Verapamil

Previous studies have demonstrated that the elevated level of cytosolic Ca^2+^ in ALS MNs is associated with autophagy regulation in a mTOR-independent manner. Verapamil is a L-type Ca^2+^ channel blocker used clinically for cardiovascular diseases. It can activate autophagy and improve autophagy influx by reducing intracellular Ca^2+^. In the SOD1^G93A^ mouse model, intraperitoneal injection with verapamil (25 mg/kg per day) could delay the disease onset and extend survival by improving autophagy influx and reducing SOD1 aggregation [99].

#### Torkinib

Stress granules play an important role in regulating the formation of insoluble aggregates in pathological conditions [100]. Recently, Torkinib, a selective mTOR inhibitor, has been demonstrated to reverse the pathological changes of stress granules in induced pluripotent stem cell (iPSC)-derived neurons with P525L *FUS* mutation, which is one of the most severe mutations of ALS [101].

#### Bosutinib and Ropinirole

Bosutinib and ropinirole are two candidate anti-ALS drugs recently identified in iPSC-based drug screens and are now under clinical investigation [102]. Bosutinib, an inhibitor of Src/c-Abl, has been found to increase the survival of ALS iPSC-derived MNs by inducing autophagy and reducing misfolded SOD1 and TDP-43 proteins. Furthermore, bosutinib has been shown to delay disease onset and prolong survival of SOD1-mutant mice [103]. A 12-week phase I dose-escalation trial has been initiated in ALS subjects (UMIN000036295) to evaluate the safety and tolerability of bosutinib at 4 levels (100, 200, 300 or 400 mg/day) [20]. Ropinirole, an agonist of dopamine D2 and D3 receptor (D2R/D3R), has shown a mitochondrion-targeted antioxidant effect in iPSC-derived ALS MNs. Recent reports have demonstrated that agonists of D2R/D3R might promote autophagy through a Beclin-1-dependent pathway [102]. ﻿It would be interesting to determine whether the ropinirole-induced D2R/D3R activation leads to degradation of abnormal RNA–protein complexes *via* autophagy activation in ALS MNs.

#### Tamoxifen

Pathologic TDP-43 accumulation is one of the typical pathophysiological manifestations in ALS. Tamoxifen has been validated to enhance autophagy through the mTOR-dependent pathway by inhibiting AKT/PKB and increase the clearance of TDP-43 aggregates. A small phase II clinical trial initiated to explore the efficacy of tamoxifen in ALS patients has been completed in Taipei (NCT02166944). Among 18 patients, 10 of them were randomly assigned to receive tamoxifen treatment (40 mg daily for 1 year). According to the result from the first 6-month follow-up, a slower decline rate of ALSFRS-R score was observed in the tamoxifen group, though there was no difference in the ALSFRS-R score between the two groups at the 12-month follow-up. This outcome demonstrated a modest effect of tamoxifen in slowing progression during 6-month follow-up. Given the small-scale of this trial, larger-scale trials are required for a robust conclusion on tamoxifen efficacy [21].

#### Histone deacetylase 6 (HDAC6)-targeting strategy

HDAC6 plays an essential role in the regulation of mutant SOD1 aggregation [104, 105] and controls the fusion of autophagosomes to lysosomes [106]. While some studies have demonstrated detrimental effects associated with HDAC6 inhibition, a recent study showed that overexpression of HDAC6 in ALS mice could prolong the lifespan by inducing the formation of autolysosomes and the degradation of mutant SOD1 protein aggregates [107]. In another study, genetic ablation of HDAC6 increases the number of remaining neurons in the ventral horn of the spinal cord, along with a significant improvement in the survival of SOD^G93A^ mice [108].

### Cell-based therapies

Accumulating evidence has supported the idea that transplantation of stem cells may become a promising alternative therapy for ALS [109, 110]. Transplanted stem cells could secrete growth factors such as glial cell-derived neurotrophic factor (GDNF), brain-derived neurotrophic factor (BDNF), vascular endothelial growth factor (VEGF), and insulin-like growth factor-1 to provide neurotrophic support and slow the degeneration of MNs [110, 111]. To date, several types of stem cell with various properties and therapeutic effects have been used in preclinical and clinical trials for ALS. Among them, neural stem/ precursor cells (NSCs/NPCs), mesenchymal stem cells (MSCs) and hematopoietic stem cells (HSCs) are most widely used.

#### NSCs/NPCs

In a recent study, clinical-grade human NSCs (hNSCs) were bilaterally transplanted into the anterior horns of the lumbar spinal cord of *SOD1* mutant rats (4 sites, L3–4 segment), and 40 days after the transplantation, the hNSCs integrated extensively within the cord, presented with neural phenotypes, and migrated from the injection site for 3.77 ± 0.63 cm. More importantly, the transplantation delayed the decrease of body weight and the deterioration of motor performance, reduced the level of astroglial and microglial activation, and increased the density of MNs [112]. In another study, transplantation of hNSCs engineered to secrete glial cell line-derived GDNF into the SOD1^G93A^ ALS rat cortex induced a delay of disease pathology and prolongation of survival without adverse effects. This demonstrates that the motor cortex could also be a transplant site in addition to the spinal cord [113]. Previous clinical studies have confirmed the safety and feasibility of intrathecal NSC transplantation [22–24]. The combined lumbar (L2–L4) and cervical cord (C3–C5) injections at 20 sites (400,000 cells/injection) have proven to be safe and well-tolerated in 15 participants without acceleration of the disease progression, which indicates that intrathecal transplantation of hNSCs can be safely accomplished at high doses (NCT01730716) [24]. Consistently, a recent phase I clinical trial (NCT01640067) using a highly standardized cell drug product has obtained the same results and underscored good reproducibility and homogeneity of stable hNSCs lines. The potential therapeutic effects of hNSCs have provided sufficient promise for future phase II trials, which are currently in preparation by the same group [25]. Once the therapeutic effect and safety of this standardized cell drug product are firmly verified, it is promising to address thorny issues related to ethics and source restrictions in the future.

#### MSCs

MSCs can be obtained from bone marrow, peripheral blood, umbilical cord blood and adipose tissue. The relative ease of in vivo harvesting and expansion of patient-derived MSCs has allowed their wide application by cell transplantation in studies of neurologic diseases with less risk of rejection and fewer ethical issues [112]. Multiple intracerebroventricular transplantation of MSCs (250,000 hUC-MSCs resuspended in 8 μl of sterile PBS) isolated from the human umbilical cord protected MNs but had no beneficial effects on the survival of ALS mice [114]. The safety and feasibility of MSC transplantation in the central nervous system have been demonstrated by a phase I clinical trial. In that trial, expanded autologous MSCs suspended in the autologous CSF were injected into the thoracic spinal cord. After transplantation, no structural or pathologic change was observed in the central nervous system (CNS) as revealed by MRI scanning [115]. Further phase I and II clinical studies have been conducted to investigate the safety of repeated intrathecal injection of MSCs from human bone marrow. Results have shown increased TGF-β and IL-10 as well as reduced MCP-1 expression, which are related to MN injury, suggesting that intrathecal injection of MSCs may modulate immunoinflammation in ALS patients [26, 27]. In the phase II study (NCT01363401), two repeated treatments with intrathecal autologous bone marrow-derived MSCs (1 × 10^6^ cells/kg with a 26-day interval) showed significant therapeutic benefits with safety in ALS patients [27]. MSCs can be induced to offer enhanced secretion of neurotrophic factors (GDNF, BDNF, VEGF), and the treated MSCs were developed as a cell product called MSC-NTF or NurOwn by BrainStorm Cell Therapeutics in 2007 [116]. In a phase I/II and a IIa clinical trials (NCT01051882 and NCT01777646), intramuscular or intrathecal administration of NurOwn is safe and well-tolerated. Furthermore, possible clinical benefits were observed in patients with intrathecal administration, or combined intrathecal and intramuscular administration [28]. Recently, a phase II RCT (NCT02017912) tested the single-dose transplantation of MSC-NTF cells, and found a higher proportion of treated participants (combined intrathecal and intramuscular administration) with ≥1.5 points/month ALSFRS-R slope improvement, increased neurotrophic factors and decreased inflammatory biomarkers after transplantation, in addition to a good safety, suggesting promising efficacy of NurOwn transplantation [29]. Another phase I/II clinical trial (NCT02286011) selected the tibialis anterior muscle as a transplant site, and found that single injection of BM-MSCs (ranging 206 × 10^6^–1086 × 10^6^ cells) was safe and caused a higher D50 index, a parameter used to quantify the compound muscle action potential scan curve which decreases with disease severity [30]. As muscle is predominantly affected in ALS, retarding the progressive loss of motor units and denervation atrophy may improve the functional outcome or survival. Therefore, direct intramuscular implantation combined with intrathecal injection may be more effective and need to be assessed in future trials.

Respiratory dysfunction is the most common cause of death in ALS patients [3, 6]. Numerous microhemorrhages caused by microvasculature impairment have been observed in SOD1^G93A^ mice at the late stage, which may cause respiratory complications in ALS [117]. Microvasculature impairment is relevant to the damage of microvessel endothelial cells. In SOD1^G93A^ mice with hemorrhagic damage in the lung, intravenous transplantation of BM-MSCs attenuates endothelium damage through the re-establishment of vascular integrity by BM-MSCs. The transplanted cells could also release VEGF, angiogenin, and vesicles to promote angiogenesis in the lung and mediate intercellular communication [117].

#### HSCs

Neuroinflammation characterized by activation of neuroglia cells and infiltration of peripheral monocytes and lymphocytes is increasingly being recognized as a key pathogenic feature of ALS [118]. Regulatory T lymphocytes (Tregs) are a subpopulation of immunosuppressive T lymphocytes, and their dysfunction has been demonstrated in ALS patients [119, 120]. HSCs are multipotent cells that have the potential to differentiate into all mature cell types in blood, and the transplantation of HSCs has been proven to suppress inflammation and modulate immune response [116, 121]. HSC transplantation has been used for clinical treatment for a long time, and the ease of collection from peripheral blood or bone marrow, together with the non-invasive nature of administration, has made it a reliable treatment for ALS [116, 122]. Transplantation of bone marrow which could reconstitute Tregs has exerted positive effects on survival in an ALS mouse model by modulating the trophic/cytotoxic balance of glia [123]. Recently, a phase I, first-in-human study (NCT03241784) found that autologous infusion of expanded Tregs was safe and well-tolerated in all three patients. After a total of 8 infusions of expanded autologous Tregs (1 × 10^6^ cells/kg) with concomitant subcutaneous IL-2 injections (once a week, 2 × 10^5^ IU/m^2^) that help enhance the proliferation and function of infused Tregs, slowing of progression rate was observed at both early and later stages of the disease. Meanwhile, the increased suppressive function of Tregs showed a positive correlation with the slowing of clinical progression, supporting the value of Tregs suppressive function as an indicator of clinical status [31].

Despite the numerous preclinical and clinical studies demonstrating safety and therapeutic benefits in ALS patients and animal models, large, prospective RCTs with long-term follow-up are still needed to identify the safety, efficacy and optimal dose of these therapies [124, 125].

### Gene therapies

Gene therapies are used to replace or correct a defective gene involved in disease pathogenesis. The latest strategies developed to suppress the expression of mutated genes include anti-sense oligonucleotides (ASOs), RNA interference (RNAi) and CRISPR-Cas9 genome editing system [126].

#### ASOs

ASOs are short oligonucleotide sequences that selectively target and bind mRNA to interfere with its processing or transduction in many different ways, thereby preventing or modifying the expression of toxic protein. ASOs can induce cleavage of RNA by activating endogenous, intranuclear RNase H or preventing the interaction with specific RNA-binding proteins, thereby modulating its splicing [127]. In 2016, nusinersen (Spinraza) for spinal muscular atrophy became the first FDA-approved ASO-based therapy for neurodegenerative diseases [128]. In a phase I trial (NCT01041222), an ASO designed to inhibit SOD1 expression (ISIS 333611) was delivered by a single intrathecal infusion (L3–4) for 11 h and 22 min using an infusion pump at increasing doses (0.15 mg, 0.5 mg, 1.5 mg, 3 mg), and results showed that it was safe and well-tolerated [32]. ASOs can also effectively decrease the expression of *SOD1* mRNA and protein in the CSF and brain of SOD1^G93A^ rat model [129]. Recently, a single dose of next-generation *SOD1* ASOs has shown a positive effect on the survival of SOD1^G93A^ rats and mice, which was prolonged by 50 days and 40 days, respectively. Furthermore, it turned out that the initial loss of compound muscle action potential could also be reversed in SOD1^G93A^ mice [130]. Hexanucleotide expansions in *C9ORF72* are a common genetic cause for ALS and frontotemporal dementia (FTD). Previous studies have shown that ASOs could suppress RNA foci formation and gene expression and abrogate the *C9ORF72* RNA expansion-dependent pathology, the aggregation of RNA-binding proteins and the glutamate-mediated excitotoxicity in fibroblasts and human-derived iPSC neurons [127, 131, 132]. In *C9ORF72* transgenic mice, cerebroventricular injection of single-dose ASOs could reduce RNA foci and dipeptide-repeat proteins, and ameliorate behavioral deficits [133]. Ataxin 2 gene is also associated with the risk of ALS. Administration of ASOs targeting ataxin 2 in the CNS of TDP-43 mice could greatly extend the survival and improve the motor performance of the mice [134].

The ASO-based therapy has shown great potential in preclinical studies. More recently, a phase I-II ascending-dose trial (NCT02623699) was conducted to evaluate the safety, pharmacokinetics and pharmacodynamics of tofersen in ALS cases carrying *SOD1* mutation [33]. Tofersen is an ASO that reduces SOD1 protein synthesis by mediating the degradation of SOD1 mRNA. In that trial, 48 participants were randomly assigned to receive different doses of tofersen (20 mg, 40 mg, 60 mg, or 100 mg) or placebo by lumbar intrathecal bolus injection on days 1, 15, 29, 57, and 85. The total SOD1 protein concentration in the CSF was reduced in the four dose cohorts. The change of CSF SOD1 concentration at day 85 from that at baseline in the tofersen groups differed from the placebo group by 2%, − 25%, − 19%, and − 33%, respectively. Moreover, in the fast-progression subgroup, the ALSFRS-R score of the 100 mg tofersen group seemed to decrease slower than the placebo group [33]. For further investigation of the safety and efficacy of tofersen, a phase III, randomized, double-blind, placebo-controlled trial (NCT02623699) and its long-term extension study (NCT03070119) are currently underway.

#### RNAi

RNAi is another approach against RNA-mediated gain of toxicity, in which long double-stranded RNA duplexes are first processed within the cell and loaded into an RNA-induced silencing complex (RISC). The binding of RISC and the targeted cellular mRNA will then silence the mRNA by RNase-mediated degradation or translational repression [135]. RNAi can be induced by small RNA duplexes, and the three most common duplexes are artificial microRNA (miRNA), short interfering RNAs, and short hairpin RNAs (shRNAs) [135]. Due to the blood-brain barrier (BBB), viral vectors derived from lentivirus and adeno-associated virus (AAV) are used for therapeutic gene delivery [136]. In an animal study, neonatal SOD1^G93A^ mice receiving injection of a single-stranded AAV9 vector encoding an artificial mRNA against human *SOD1* in the cerebral lateral ventricles showed improvements in multiple parameters including survival, number of MNs, the extent of neuroinflammation, diameter of ventral root axons and pulmonary function [137]. Similar results have been demonstrated in another study [138]. A recent study has demonstrated that the AAV5-delivered artificial miRNAs targeting *C9orf72* could reduce the accumulation of repeat-containing *C9orf72* transcripts in both iPSC-derived neurons and ALS mouse model [139, 140]. Although an allele-specific silencing effect has been observed through intrathecal or intraventricular delivery of the vector, there are still some limitations in the delivery method. To improve the penetrability of the viral delivery vectors, high titers are required, which limits the feasibility, effectiveness, and safety of this gene silencing therapy. An excellent work using a spinal subpial method to deliver AAV9-mediated shRNA targeting SOD1 has provided a potent approach for transduction of neurons and surrounding glia and showed higher efficiency in reducing *SOD1*^*G37R*^-encoding mRNA than the intrathecal injection method [141]. Additionally, the spinal subpial delivery method shows an impressive effect in delaying disease onset and stopping disease progression in *SOD1*^*G37R*^ mice [141]. Therefore, seeking appropriate delivery methods should also be viewed as an important part of future studies.

The safety and efficacy of RNAi-based therapies has been tested predominantly in preclinical models of ALS. Recently, two ALS patients with *SOD1* mutation were treated with a single intrathecal infusion of AAV encoding a miRNA targeting SOD1 [142]. Unfortunately, patient 1 died from respiratory failure 15.6 months after the initiation of treatment. Autopsy results of patient 1 revealed lower SOD1 levels in the spinal cord than in the untreated patients. In addition, transient and slight reduction of SOD1 levels in the CSF was observed in patient 1. Although having no change in the CSF SOD1 level, the patient 2 had stable functional status and vital capacity for 12 months [142]. In general, this study demonstrated that intrathecal miRNA might be a promising treatment for ALS. However, due to the heterogeneity of ALS and the insufficient sample size, confirmative conclusions on treatment effects could not be drawn.

#### CRISPR-Cas9 genome-editing system

The CRISPR/Cas9 genome editing system is emerging as a promising tool to disrupt the expression of mutant genes at the genomic level. CRISPR/Cas9 is a type II CRISPR/Cas system with guide RNA to target specific DNA sequences and with Cas9 as a nuclease [143]. CRISPR/Cas9 has already been successfully used to establish gene-corrected ALS iPSCs [144]. Moreover, when delivered by the AAV vector in SOD1^G93A^ mice, CRISPR/Cas9 could disrupt expression of mutant SOD1 and reduce the protein level of mutant SOD1 in the spinal cord, accompanied by prolonged survival and improved motor function [126, 143]. RNA-targeting Cas9 has been reported to be able to reduce RNA foci and polyglutamine protein products in the *C9orf72*-linked ALS patient cells [145]. In the *C9orf72*-linked ALS cell line, transcriptional inhibition mediated by deactivated Cas9 could rescue the splicing defects and block the repeat-associated non-ATG translation to reduce toxic dipeptide polymers [146]. In addition, increased expression of the GluA1 AMPA receptor (AMPAR) subunit observed in iPSC-derived MNs with *C9ORF72* mutations, but not in iPSC-derived cortical neurons, is rescued by CRISPR/Cas9-mediated correction of the *C9ORF72* repeat expansion in MNs, together with the abolishment of increased Ca^2+^-permeable AMPAR expression and MN vulnerability to excitotoxicity caused by the increased GluA1 subunit [130]. Andrade et al. have reported that dipeptide repeat proteins (DPRs) encoded by *C9ORF72* through a non-canonical translation mechanism are neurotoxic and can increase the frequency of DNA double-strand breaks (DSBs) by inhibiting the key DNA DSB repair pathways. It is noteworthy that the accumulation of DNA DSBs is reduced and the dysregulation of single-strand annealing is improved after deletion of *C9ORF72* expansion by CRISPR/Cas9 in C9ALS iPSC lines [147]. These results indicate that genome editing is the best approach to correcting disease-causing mutations. However, the safety, target specificity, immunogenicity as well as ethical concerns need to be addressed before translation to clinical use.

#### Limitations of gene therapies for ALS

ALS is a lethal neurodegenerative disorder without effective treatment. Heritability research has demonstrated that about 60% of the risk of ALS is genetically determined [9, 38] and mutations in more than 25 genes have been found in patients with or without a family history [148, 149]. In recent years, the development of gene therapy techniques and clinical application of the nusinersen-based therapy targeting spinal muscular atrophy have demonstrated great potentials of gene therapies in treatment of neurological disorders, suggesting that the gene editing approach may hold promise for ALS treatment. To date, applications of gene therapies in ALS have mainly focused on *C9ORF72* correction. *C9ORF72* repeat expansion is identified as the most common genetic cause for ALS and FTD. Abnormal translation of the expanded repeat will result in loss of function, RNA toxicity, and DPR protein toxicity that are implicated in pathogenesis, thus gene-editing techniques targeting causal mutations to cut repeat expansions, inhibit transcription, or selectively reduce the repeat-containing RNAs might be promising in retarding disease progression. The effectiveness of the ASO and RNAi therapies in reducing RNA foci has been observed in vitro and in vivo [127, 131, 139, 140]. Based on these results, a phase I clinical trial of ASOs targeting the sense strand of *C9ORF72* in C9 ALS patients has been conducted (NCT03626012). In addition, genetic correction of *C9ORF72* repeat expansions has been seen in patient iPSCs [130, 147]. These encouraging results suggest that gene editing is a promising tool for treating ALS patients carrying a single gene mutation. However, previous reports of oligogenic inheritance in ALS indicate that the early-onset patients may carry more than one ALS gene mutation [150]. In this situation, the effect of gene therapies targeting an individual mutation might become suboptimal. More importantly, the genetic heterogeneity of ALS and the limited numbers of identified genes in sALS patients make the personalized gene therapy more challenging and it is impossible to design clinical trials targeting each specific gene. However, the genetic mutations can be categorized into several subgroups that share a common mechanism of pathology, providing an opportunity for therapies. On the other hand, in the loss-of-function ALS subgroups, gene delivery strategies aiming to normalize the function of the mutated protein may be within reach. In addition, safety issues such as off-target effects and inactivation of the normal gene copy remain to be solved before clinical use. Currently, there is no effective treatment for ALS, and the inspiring results from ASOs and RNAi-based therapies in both preclinical and clinical studies are raising hope for ALS treatment.

### Others

#### Mito Q

Mito Q is an antioxidant that has been shown to mitigate oxidative damage in neurodegenerative disease models. Mito Q can accumulate within mitochondria and improve mitochondrial function in different neuronal cells exposed to oxidative stress [151]. Mitochondrial dysfunction is involved in the pathogenesis of ALS, and impaired mitochondrial function has been observed in the SOD1 mouse model. Previous studies have shown that pretreatment of SOD1^G93A^ astrocytes with Mito Q prevents the toxicity of mutant SOD1 and improves ATP generation in MNs [39, 152]. Moreover, Mito Q orally administered (500 μM) in SOD1^G93A^ mice since the presentation of early symptoms significantly preserves the neuromuscular junctions and increases the strength of hindlimbs by improving mitochondrial function and decreasing nitroxidative damage in the lumbar spinal cord and the quadriceps muscle. The extension of lifespan suggests that administration of Mito Q may slow the disease process [153].

#### Methylcobalamin

Methylcobalamin, physiologically equivalent to vitamin B12, shows a protective effect against glutamate-induced cytotoxicity in cultured cortical neurons [154]. Multivitamin therapy with vitamin B12 and folic acid significantly delays disease onset and prolongs the average lifespan of SOD1^G93A^ transgenic mice [155]. In addition, a retard in the progression of motor symptoms and neuropathological changes has been observed in wobbler mice after intraperitoneal treatment with high-dose methylcobalamin [156]. In a recently reported phase II/III clinical trial (NCT00444613) on the safety and efficacy of intramuscular ultra-high-dose methylcobalamin in ALS patients, primary endpoints of survival and ALSFRS-R change both failed to show a difference between methylcobalamin (25 and 50 mg) and placebo groups. However, post-hoc analyses demonstrated that 50 mg methylcobalamin prolonged survival and retarded symptomatic progression when administered at an early stage (≤12 months after symptom onset) [34].

#### Rasagiline as an add-on therapy to riluzole

Rasagiline, a monoamine oxidase B inhibitor with a symptomatic efficacy in Parkinson’s disease, prolongs survival in preclinical animal studies of ALS, both alone and in combination with riluzole [157]. In a small US study with 80 ALS patients and 177 historical placebo controls, rasagiline (2 mg/day) alone neither altered disease progression nor showed evidence for biomarker engagement [35]. Albert Ludolph and colleagues conducted a phase II RCT (NCT01879241) of rasagiline (1 mg/day) as an add-on treatment to riluzole in 252 ALS patients. No difference in the primary outcome of survival was observed between the rasagiline and placebo groups. However, post-hoc stratifications revealed a possible survival benefit and slower functional decline in a subset of patients with an initial slope of ALSFRS-R greater than 0.5 points per month [36].

#### Masitinib as an add-on therapy to riluzole

Masitinib, a targeted anticancer drug, has shown therapeutic potential in SOD1^G93A^ mice *via* its immunomodulatory properties targeting microglia as well as macrophage activity in both CNS and peripheral nervous system. The efficacy of masitinib as an add-on therapy to riluzole has been assessed in a phase II/III clinical trial over a 48-week treatment period (NCT02588677), in which 394 ALS patients were randomly assigned to receive riluzole (100 mg/day) plus placebo or masitinib (3.5 or 4.5 mg/kg per day). Compared with the placebo group, riluzole plus masitinib (4.5 mg/kg per day) showed a significant 27% slowing in rate of functional decline [37]. Significant results were also found for secondary endpoints such as ALSAQ-40, FVC and time-to-event analysis [37].

#### AMX0035

AMX0035, a combination of sodium phenylbutyrate and ﻿taurursodiol, ﻿was designed to attenuate neuronal death by mitigating ER stress and bioenergetic dysfunction. In a multicenter, phase 2, placebo-controlled trial (CENTAUR) that evaluated the efficacy of oral AMX0035 in ALS patients, ﻿there was a significant difference of 0.42 points per month between the active-drug group and the placebo group in the mean rate of change in the ALSFRS-R total score over 6 months, representing an approximately 25% slowing of disease progression [158]. In the open-label extension of CENTAUR, random assignment to AMX0035 at baseline resulted in a 6.5-month survival advantage compared with the placebo assignment [159].

### Nanotechnology-based strategies

Although a wide variety of therapeutic agents have proven effective in ALS preclinical studies and some are undergoing clinical investigation, their efficacy is still suboptimal and far from satisfactory due to the challenges regarding safe and effective delivery routes. These challenges can be attributed to the BBB/blood-spinal cord barrier, as well as insufficient biostability/bioavailability and “off-target” effects. Encouragingly, the development of nanotechnology-based strategies allows for the improvement of therapies in, e.g., effective delivery of drugs, genes and ASOs to the CNS, and promoting the effectiveness of stem cell therapies [160, 161].

For example, Bondi and collaborators have employed solid lipid nanoparticles as a drug carrier for riluzole, and found that riluzole carried by solid lipid nanoparticles accumulates less in the nontarget organs [162]. Besides, the concentration of riluzole tested in the rat brain is significantly higher when using solid lipid nanoparticles as a delivery system [162]. Shashi et al. encapsulated riluzole in tween80-coated, chitosan-conjugated N-isopropylacrylamide nanoparticles and found that the nanoriluzole could effectively cross the BBB and exhibit neuroprotective effect by reducing the expression of inflammatory molecules and increasing the glutathione level at a very low concentration [163]. Liposomes, also known as liposomal nanoparticles, have been employed by Yang et al. to overcome the pharmaco-resistance problem and improve the transportation of riluzole in the CNS. They found that the verapamil and riluzole cocktail liposomes could suppress the function of efflux transporters and improve the uptake of riluzole in an in vitro BBB cell model [164]. Similar effects have been achieved for edaravone encapsulated into agonistic micelles [165].

Therapies targeting gene defects, such as the RNAi and ASOs technologies, are greatly limited by the delivery barriers. AAV and lentivirus are the most common carriers used in present studies; however, concerns on safety, immunogenicity, expression time of exogenous genes as well as the package and isolation of the virus have interfered with their clinical use and therapeutic effect. In 2017, calcium phosphate lipid-coated nanoparticles (CaP-lipid NPs) were developed for the delivery of *SOD1* ASO to MNs [166]. The particles have an encapsulation efficiency of 48% for ASO and remain stable for more than 20 days. The CaP-lipid NPs can effectively deliver SOD1-targeting ASOs into the NSC-34 cell line and suppress *SOD1* expression in HEK293 cells [166]. Moreover, the CaP-lipid NPs can be efficiently delivered to the CNS of zebrafish and have a prolonged circulation time within the bloodstream, which indicates that nanoparticles may become an ideal delivery system for gene therapy [166].

Biomaterials can also be used in stem cell-based therapies to provide a more permissive microenvironment for stem cell growth and distribution, and allow visualization of intrathecal delivery and targeted cell placement process. The use of high-speed MRI could track the stem cell infusion process in real-time when MSCs are labeled by superparamagnetic iron oxide nanoparticles, which can ensure placement of stem cells in defined brain regions and avoid formation of potentially dangerous cell aggregates [167]. Besides nanoparticles, hydrogels have also demonstrated potentials as cell delivery vehicles and imaging probes in ALS treatment [168, 169]. Human-derived adipose stem cells encapsulated in formulations of methacrylated gellan gum/hyaluronic acid hydrogel blends could stay in vitro for over 14 days and be visible as a hyperintense signal in T1 MRI for 24 h after transplantation in vivo [169]. Hydrogel exhibits great strengths in improving biodistribution and providing physical support for stem cells to enhance their survival, which makes it a promising tool for injectable image-guided cell delivery approaches [168, 169].

## From bench to bedside: bottlenecks in translating preclinical studies in ALS and future directions

﻿ Great efforts have been made in search for effective therapies for ALS throughout the years. Based on the promising results in preclinical cellular and animal models, hundreds of agents have been proposed as candidates for ALS treatment. However, clinical trials have predominantly come to disappointing results in humans. ﻿Several obstacles are implicated in the failure of translation from bench to bedside in the field of ALS treatment.

First, the inadequacy of ALS models is considered as an important reason for failed clinical trials. At the preclinical level, genetically modified rodents carrying fALS mutations remain the most widely used models. However, they do not fully recapitulate the complete pathophysiological and phenotypic spectrum present in ALS patients. ALS is caused by defects in different interacting pathways that culminate in a large network. ﻿The relative extent to which each of these mechanisms contributes to the overall pathobiology of ALS has not been fully ascertained, making it difficult to discriminate initiating factors from secondary consequences and target the primary processes underlying ALS. A more comprehensive understanding of ALS pathogenic mechanisms will allow for advances in treatment.

Second, the lack of presymptomatic biomarkers and the delay in clinical diagnosis have significantly limited the therapeutic potential of putative disease-modifying drugs. It is now increasingly accepted that by the time patients fulfil the diagnostic criteria for ALS, a considerable disease burden has already occurred in the long presymptomatic phase. The timing of application of potentially neuroprotective interventions is fundamental for increasing the chance of success. The results of treatment that was started much in advance with respect to the onset of symptoms in animal models should be considered as indicative of success in humans. Pre-symptomatic diagnosis and clinical trials of early therapeutic intervention in fALS patients identified to carry ALS-related gene mutations are strongly recommended in the future. Also, potential biomarkers that allow early intervention to improve therapeutic outcomes of ALS are anticipated in the near future.

## Conclusions and comments

There is an urgent need to develop novel disease-modifying therapeutics to slow the disease progression and extend the lifespan of ALS patients. Since emerging evidence has supported the notion that dysregulation of autophagy is critical for the pathogenesis of ALS, the autophagic signal pathway may be a potential therapeutic target. Furthermore, studies elucidating the genotype–phenotype correlations in ALS patients in recent years have laid ground for individualized, gene-specific therapeutic approaches. The results of these studies advance our understanding of ALS and boost our hope that disease progression can be curbed in the future.

## Data Availability

All the data mentioned in this article are available in published article.

## Abbreviations

ALS: Amyotrophic lateral sclerosis
MNs: Motor neurons
fALS: Familial amyotrophic lateral sclerosis
sALS: Sporadic amyotrophic lateral sclerosis
SOD1: Superoxide dismutase 1
*C9orf72*: Chromosome 9 open reading frame 72
TARDBP/TDP43: TAR DNA-binding proteins
ER: Endoplasmic reticulum
ALSFRS-R: Amyotrophic Lateral Sclerosis Functional Rating Scale Revised
mTOR: Mammalian target of rapamycin kinase
HDAC6: Histone deacetylase 6
GSK-3β: Glycogen synthase kinase-3β
Atg: Autophagy-related protein
LC3: Microtubule-associated protein 1A/1B-light chain 3
PE: Phosphatidyl ethanolamine
TFEB: Transcription factor EB
LAMP1: Lysosomal-associated membrane protein 1
Rab7: Ras-related protein 7
GDNF: Glial cell-derived neurotrophic factor
BDNF: Brain-derived neurotrophic factor
VEGF: Vascular endothelial growth factor
NSC/NPCs: Neural stem/ precursor cells
MSCs: Mesenchymal stem cells
HSCs: Hematopoietic stem cells
CNS: Central nervous system
ASOs: Anti-sense oligonucleotides
RNAi: RNA interference
CSF: Cerebrospinal fluid
PSC: Induced pluripotent stem cell
RISC: RNA-induced silencing complex
miRNA: MicroRNA
shRNAs: Short hairpin RNAs
AAV: Adeno-associated virus
AMPAR: AMPA receptor
DPRs: Dipeptide repeat proteins
DSBs: Double-strand breaks
BBB: Blood-brain barrier
CaP-lipid NPs: Calcium phosphate lipid-coated nanoparticles
